# Spatial-Intensity Transforms for Medical Image-to-Image Translation

**DOI:** 10.1109/TMI.2023.3283948

**Published:** 2023-10-27

**Authors:** Clinton J. Wang, Natalia S. Rost, Polina Golland

**Affiliations:** Computer Science and Artificial Intelligence Laboratory, MIT, Cambridge, MA 02139 USA; Department of Neurology, Massachusetts General Hospital, HMS, Boston, MA 02114 USA.; Computer Science and Artificial Intelligence Laboratory, MIT, Cambridge, MA 02139 USA

**Keywords:** Spatial-intensity transform, image-to-image translation, generative adversarial network, longitudinal image prediction, counterfactual image generation

## Abstract

Image-to-image translation has seen major advances in computer vision but can be difficult to apply to medical images, where imaging artifacts and data scarcity degrade the performance of conditional generative adversarial networks. We develop the spatial-intensity transform (SIT) to improve output image quality while closely matching the target domain. SIT constrains the generator to a smooth spatial transform (diffeomorphism) composed with sparse intensity changes. SIT is a lightweight, modular network component that is effective on various architectures and training schemes. Relative to unconstrained baselines, this technique significantly improves image fidelity, and our models generalize robustly to different scanners. Additionally, SIT provides a disentangled view of anatomical and textural changes for each translation, making it easier to interpret the model’s predictions in terms of physiological phenomena. We demonstrate SIT on two tasks: predicting longitudinal brain MRIs in patients with various stages of neurodegeneration, and visualizing changes with age and stroke severity in clinical brain scans of stroke patients. On the first task, our model accurately forecasts brain aging trajectories without supervised training on paired scans. On the second task, it captures associations between ventricle expansion and aging, as well as between white matter hyperintensities and stroke severity. As conditional generative models become increasingly versatile tools for visualization and forecasting, our approach demonstrates a simple and powerful technique for improving robustness, which is critical for translation to clinical settings. Source code is available at github.com/clintonjwang/spatial-intensity-transforms.

## I. Introduction

Image-to-image translation, which maps images in one distribution to images in another distribution, is a common task in computer vision and medical image analysis. In medical contexts, conditional generative adversarial networks (GANs) that change an input image along a set of controlled attributes (e.g., imaging modality or patient phenotype) are useful in a wide range of applications including CT denoising [1], [2], data augmentation [3], super-resolution [4], [5], MRI reconstruction [6], [7], CBCT reconstruction [8], MR-to-CT translation [9], and prediction of disease trajectories [10]. However, such models may introduce artifacts when trained on small or noisy datasets, particularly with lower quality clinical scans. Artifacts like bright/dark spots or blurred tissue can appear similar to biomarkers of disease, and thus a generative model that causes such artifacts may frequently create misleading outputs.

We address this shortcoming by introducing a regularized parameterization of the generator called a spatial-intensity transform (SIT). Instead of outputting a new image directly, a SIT generator outputs a smooth deformation field (*i.e*. diffeomorphism) and sparse intensity difference map which are then applied to the input image. SIT is a simple, fast network component that can be readily applied to a wide range of models without adding learnable parameters. We employ SIT models to predict future brain scans of patients with neurodegenerative disease and to visualize counterfactual brain scans in a cohort of patients with acute ischemic stroke conditioned on age and disease severity. On both tasks, we show that SIT networks produce anatomically plausible output images with fewer artifacts than their unconstrained versions.

### A. Prior Work

#### 1) Spatial and Intensity Transforms:

Spatial transforms have a rich history in medical image registration. Nonrigid transformations are often represented as a smooth deformation field since most anatomical variation does not involve large local changes in shape. The transform can be optimized independently for each input image using some similarity measure such as mutual information [11], [12]. Parameterizing the space of deformations using diffeomorphisms gives rise to Large Diffeomorphic Distance Metric Mapping (LDDMM) [13], Diffeomorphic Anatomical Registration using Exponentiated Lie algebra (DARTEL) [14], and symmetric diffeomorphic image registration with cross-correlation ] [15]. Recent data-driven approaches train a neural network to generate a deformation field or the velocity field of a diffeomorphism [16], [17]. Our work uses the SIT generator to produce a diffeomorphic deformation in a similar manner.

Spatial transforms have been coupled with intensity transforms to perform image registration when there is variation in both anatomy and texture. Active Appearance Models were used to build statistical models of shape and intensity that can be used to register images with different tissue intensities [18]. Data-driven applications of spatial-intensity transforms enable atlas building in the presence of pathology [19] as well as the construction of atlases conditioned on age [20]. Spatial-intensity transforms have also been featured in data augmentation techniques for few-shot [3] or one-shot [21] segmentation. Depending on the application, the intensity transform can be sparse to represent localized phenomena, smooth to represent diffuse tissue changes, or explicitly designed to reflect anatomical priors about well-understood biological phenomena [10].

Spatial and/or intensity transforms have also been used to produce more robust or interpretable generative models. The closest work to ours uses a conditional GAN parameterized by only spatial transforms to highlight biomarkers of Alzheimer’s disease in brain MRIs and chronic obstructive pulmonary disease in chest x-rays [22]. However, this parameterization is unable to capture changes in intensity, and hence the model can only describe morphological biomarkers. Spatial-intensity transforms have been applied to translate scans across clinical sites for multi-site harmonization, where the spatial component permits visual inspection of the deformation field for plausibility [23].^1^ In this work, we develop a general, streamlined implementation of spatial-intensity transforms that retains this visualization capability while producing high-fidelity images. We also extend the use of spatial-intensity transforms to the task of medical image-to-image translation conditioned on arbitrary attributes.

#### 2) Image-to-Image Translation:

Conditional generative adversarial networks (cGANs) provide a powerful data-driven method for performing image-to-image translation tasks, achieving state of the art results in applications as diverse as sketch to photo conversion [24], image colorization [25], image inpainting [26], and style transfer [27]. Like other GANs, cGANs train a generator and discriminator adversarially – the discriminator is trained to distinguish real images in the dataset from synthetic images produced by the generator, while the generator is trained to fool the discriminator [28]. When the training data contains paired images showing the desired translation between two domains, the generator can be trained to match ground truth images, using a pixel-wise L1 loss for example.

However, paired training data is often impossible to obtain. In such cases, one approach is to teach the generator to project images to a latent space, update the latent vector with information about the desired translation, and then decode this new latent vector to produce a translated image. This method is used by conditional adversarial autoencoders [29] and the identity-preserving GAN [30]. Another approach is to build a model that learns both forward and inverse maps between domains, and makes the composition of the forward and inverse generators close to identity using a cycle consistency loss. This method is used by CycleGAN [31], which translates images between two domains, and StarGAN [32], which translates images between an arbitrary number of domains by using a classifier to guide the generator to produce images belonging to the desired domain. We extend this technique to translate images along multiple continuous attributes by using a regressor. Our implementation also builds on the observation that a generator parameterized by the difference between source and target attributes rather than the raw target values tends to better preserve unchanged attributes [33]. We show that this approach has the added benefit of learning from datasets with partially labeled attributes – a common phenomenon in medical datasets.

#### 3) Artifacts in Generative Models:

Even GANs that produce perceptually convincing outputs can introduce subtle artifacts into their images [34], [35]. Several works have probed why unconditional GANs can generate artifacts in the context of natural images. Using deconvolution layers leads to checkerboard artifacts, and pixel-wise normalization can result in mismatched colors in RGB images [36]. In StyleGAN, adaptive instance normalization layers create blob-shaped artifacts, and progressive growing causes phase artifacts [34]. Other work establishes that artifacts also appear when such models are naively applied to medical imaging contexts [37], which we find particularly true when applied to real clinical data.

Several previous works developed strategies for applying cGANs to medical images. In the context of brain aging, one strategy for making such models more robust is to incorporate prior knowledge of how age affects the intensity of each anatomical region [10]. Alternatively, an identity preservation regularization term can be used to encourage small changes in age to produce small changes in image intensity [30]. These two approaches tailor their loss functions to capture priors about the particular translation task of interest, and may not be suitable for conditional variables other than age. In contrast, spatial-intensity transforms naturally capture medical image transformations for a wide range of tasks, image modalities, and conditioning attributes, making our approach much more flexible. Since SIT only modifies a small part of the generator network, it can also be freely combined with these other strategies as we demonstrate later.

#### 4) SIT-GAN:

This paper significantly expands on the preliminary work presented in [38]. Whereas our previous work focused on applying spatial-intensity transforms to a single model derived from StarGAN, we now present spatial-intensity transforms as a general framework for improving the robustness of diverse medical image-to-image translation models. Additionally, we expand the review of prior work and perform extensive experimental evaluations. Here we provide validation against ground truth scans by evaluating the model’s ability to predict longitudinal trajectories. Furthermore, the previous work found that the spatial-intensity transform improves the quality of output images at the cost of poorer target domain transfer. Here we demonstrate that this tradeoff vanishes when the class of spatial transforms is further constrained to diffeomorphisms, which no longer need smoothness regularization. SIT-GAN is similar to SIT-Disp in our ablation experiments.

### B. Our Contributions

We demonstrate that parameterizing conditional GANs in terms of spatial-intensity transforms improves image fidelity and robustness to artifacts in medical image-to-image translation tasks, while preserving the network’s ability to match the target domain. We compare four types of image-to-image translation models, and demonstrate that spatial-intensity transforms uniformly improve the performance of these models across two different tasks. The first task predicts longitudinal brain scans of patients with various stages of neurodegenerative disease in the Alzheimer’s Disease Neuroimaging Initiative (ADNI) dataset (adni.loni.usc.edu). Here we drastically improve performance on prediction of aging trajectories in T1-weighted brain MRIs. Without supervised training on paired scans, our model accurately forecasts longitudinal brain scans of subjects with various stages of neurodegeneration.

The second cohort consists of clinical quality MRIs from patients with acute ischemic stroke from the MRI-GENetics Interface Exploration (MRI-GENIE) study [39]. By conditioning on age and disease severity, our models highlight the expansion of the ventricles associated with aging, as well as the growth in white matter hyperintensities associated with stroke severity. This experiment involves clinical quality scans of stroke patients from multiple sites and demonstrates our method’s robustness to low quality scans and its ability to generalize to unseen scanners.

The paper is organized as follows. In the next section, we introduce SIT (spatial-intensity transforms) as our parameterization of the conditional generator. We describe the four image-to-image translation models that we build on for our task, including their network architecture, loss functions, and training schemes. In Section III, we describe our experiments, including the data, evaluation metrics, and an ablation study. In Section IV, we present the results from each experiment, and discuss their implications and applications in Section V. In Section VI, we conclude that the spatial-intensity transform is a simple and effective technique for medical image-to-image translation tasks.

## II. Methods

We first describe the general problem setup for image-to-image translation. Then we present our parameterization of the generator as a diffeomorphism composed with a sparse intensity difference transform. We detail several different models for image-to-image translation, each of which we can readily adapt to use spatial-intensity transforms. Finally, we provide details of the network architecture and training.

### A. Image-to-Image Translation

Let 𝒳 be the space of images and 𝒴 be the space of conditional attributes (e.g., age and disease severity). We denote an image as a map from the space of pixel coordinates Ω to intensities: x∈𝒳:Ω→R. Here we consider continuous vector attributes $y=\left(y_{1},\ldots ,y_{m}\right)$, which may have missing data. Given a dataset of image-attribute pairs $𝒟={\left\{\left(x_{i},y_{i}\right)\right\}}_{i=1}^{N}$, we train a generator to produce a new image conditioned on an input image and a set of changes in attribute values, G:𝒳×𝒴→𝒳. We sample minibatches of image-attribute pairs (x,y) and a new set of attributes $\tilde{y}$ from the dataset. We parameterize the generators with the difference $G(x,\tilde{y}-y)$ rather than $G(x,\tilde{y})$, as this difference can be computed even when datapoints have missing conditional attributes, by using the convention that ${\tilde{y}}_{k}-y_{k}=0$ if the kth attribute is missing. The generator’s goal is to output an image whose attributes appear to take on the specified values, while preserving aspects of the input image that are unrelated to the conditional attributes, such as non-pathological anatomy.

### B. Spatial-Intensity Transforms

Typically, generators output the translated image directly after the last convolutional layer of the network. In contrast, we propose to parameterize the output of the generator using spatial-intensity transforms. For image dimensionality d, we define the outputs of the last generator layer as a stationary velocity field $V\;:\Omega \to R^{d}$ and intensity difference map Δx:Ω→R.

A stationary velocity field is a common parameterization of diffeomorphic spatial transforms, as it can be efficiently integrated via the scaling and squaring technique to produce a smooth deformation $\Phi_{V}\;:𝒳\to 𝒳$ [40]. The generator then transforms the input image x into the output image $\Phi_{V}(x+\Delta x)$. Outputs V and Δx can themselves be visualized to provide a disentangled view of the spatial and intensity transforms.

In addition, a L1-norm regularization term is added to the generator’s loss function to encourage the intensity difference map to be sparse:

(1) $$\|\Delta x{\|}_{1}=\frac{1}{\left|\Omega \right|}\sum_{\omega \in \Omega }|\Delta x(\omega )|.$$

The sparse intensity transform is designed to capture intensity changes in small regions of the image, such as focal pathology, while the diffeomorphic spatial transform captures morphological changes in anatomical structures. Note that SIT only appends a parameter-free layer to the generator network, making it a lightweight and generalizable network component that can be applied to many different models, including the four we present in the following section.

### C. Models for Image-to-Image Translation

We develop four medical image-to-image translation models, the last three of which are adapted to our setting from existing frameworks.

#### 1 Regressor-Guided Autoencoder:

The regressor-guided autoencoder (RGAE) trains the generator alongside a regressor R:𝒳→𝒴 that predicts the attributes associated with an image.^2^ The regressor is trained on real image-attribute pairs using a mean squared error loss:

(2) $${ℒ}_{R}=\frac{1}{m}E_{x,y}\left[\|R(x)-y{\|}_{2}^{2}\right],$$

where we let $(R(x)-y{)}_{k}=0$ if $y_{k}$ is missing. Meanwhile, the generator is updated using a cycle consistency loss:

(3) $$\ell_{cc}(x,y,\tilde{y})=∥G(G(x,\tilde{y}-y),y-\tilde{y})-x{∥}_{1},$$

and a relative attribute loss:

(4) $$\ell_{attr}(x,y,\tilde{y})=\frac{1}{m}∥(R(G(x,\tilde{y}-y))-R(x))-(\tilde{y}-y){∥}_{2}^{2},$$

The overall generator loss is:

(5) $${ℒ}_{G}=E_{x,y,\tilde{y}}\left[\ell_{attr}+\lambda_{cc}\ell_{cc}\right],$$

where we choose $\lambda_{cc}=0.1$. Since these terms depend on the difference between $\tilde{y}$ and y rather than their individual values, they permit missing attributes.

#### 2) Conditional Adversarial Autoencoder [29]:

The conditional adversarial autoencoder (CAAE) uses a discriminator to achieve more realistic outputs than is possible with only regressor guidance. CAAE has a generator that consists of an unconditional encoder and a conditional decoder. The encoder projects an input image to a latent vector, and the decoder produces a new image from this latent vector and a change in attributes $\tilde{y}-y$.

The generator has a reconstruction loss term:

(6) $$\ell_{rec}(x)=∥G(x,0)-x{∥}_{1}.$$

The generator is also trained alongside a conditional discriminator D:𝒳×𝒴→R (logits) that learns to assign high probability to true images with the correct attributes.

Using the Wasserstein GAN losses, the generator’s adversarial loss term is:

(7) $$\ell_{adv}(x,y,\tilde{y})=-D(G(x,\tilde{y}-y)),$$

and the discriminator is simultaneously trained with the loss: [41]:

(8) $$\begin{array}{c} {ℒ}_{D}=E_{x,y,\tilde{y}}[D(G(x,\tilde{y}-y))]-E_{x}[D(x)]\\ \;+\lambda_{GP}E_{\overset{‾}{x}}[{\left({∥\nabla_{\overset{‾}{x}}D(\overset{‾}{x})∥}_{2}-1\right)}^{2}], \end{array}$$

where $\overset{‾}{x}$ is an interpolation of real and translated images, and $\lambda_{GP}=1$ is the gradient penalty weight.^3^

An additional discriminator is imposed on the latent space produced by the encoder. The encoder is adversarially trained to produce a distribution of vectors that is close to some specified prior distribution (we use a uniform distribution over [0, 1]^50^, similarly to [29]).

With CAAE’s original loss function, we find that the discriminator ignores the conditional attributes given to it. We add loss terms that drive the discriminator to produce higher probabilities for images with the correct attributes, and drive the generator to translate images to the desired attributes:

(9) $${ℒ}_{D}^{*}={ℒ}_{D}+\lambda_{\text{cond}}\left[D\left(x,\tilde{y}\right)-D\left(x,y\right)\right],$$

where we choose $\lambda_{\text{cond}}=1$. We define

(10) $$\ell_{\text{cond}}(x,y,\tilde{y})=D(G(x,\tilde{y}-y),y)-D(G(x,\tilde{y}-y),\tilde{y}),$$

and the overall generator loss becomes:

(11) $${ℒ}_{G}=E_{x,y,\tilde{y}}\left[\ell_{adv}+\ell_{adv-z}+\lambda_{\text{cond}}\ell_{\text{cond}}\right]+E_{x}\lambda_{\text{rec}}\ell_{\text{rec}},$$

where $\ell_{adv-z}$ is the adversarial loss on the latent space and we choose $\lambda_{\text{rec}}=0.1$.

By imposing structure on latent space via adversarial training, CAAE prevents mode collapse and can represent the full distribution of images.

#### 3) Identity-Preserving GAN [30]:

Rather than relying on structured latent space, the identity-preserving GAN (IPGAN) uses an identity-preserving regularization term in the generator loss to prevent it from excessively distorting input images. The identity-preserving term penalizes the distance between input and translated images, scaling inversely with the distance between the true age $y_{0}$ and the desired age ${\tilde{y}}_{0}$. It thus enforces the prior that small differences in attributes such as age should not be accompanied by large changes in the image:

(12) $$\ell_{ID}(x,y,\tilde{y})=∥G(x,\tilde{y}-y)-x{∥}_{2}^{2}e^{-\left|y_{0}-{\tilde{y}}_{0}\right|}.$$

The generator loss is:

(13) $${ℒ}_{G}=E_{x,y,\tilde{y}}\left[\ell_{adv}+\lambda_{ID}\ell_{ID}++\lambda_{\text{cond}}\ell_{\text{cond}}\right]+E_{x}\lambda_{\text{rec}}\ell_{\text{rec}},$$

with $\lambda_{ID}=0.1,\;\lambda_{rec}=1$ and $\lambda_{cond}=1$. To save memory, we split the reconstruction loss from the other losses and train each minibatch on one set of losses at random. The discriminator loss is the same as Equation (9).

#### 4) StarGAN [32]:

In our StarGAN-derived model, the generator is trained alongside an unconditional discriminator D:𝒳→R as well as a regressor R:𝒳→𝒴 that predicts the attributes associated with an image. The combination of regressor and discriminator guidance is designed to achieve realistic outputs that match the appearance of target attributes.

The generator is updated using the Wasserstein adversarial loss of Equation (7), the cycle consistency loss of Equation (3), and the relative attribute loss of Equation (4):

(14) $${ℒ}_{G}=E_{x,y,\tilde{y}}\left[\ell_{adv}+\lambda_{cc}\ell_{cc}+\lambda_{attr}\ell_{attr}\right],$$

where $\lambda_{\text{attr}}=10$ and $\lambda_{cc}=0.1$ are empirically determined weights.

The regressor is trained to predict the attributes of real images as in RGAE (Equation (2)), and the discriminator is trained to distinguish images as in WGAN-GP (Equation (8)). We share layers between the discriminator and regressor, so a single optimizer is assigned to both subnetworks and updated using ${ℒ}_{D}+\lambda_{R}{ℒ}_{R}$ where we choose $\lambda_{R}=10$.

### D. Architecture and Implementation Details

In each model, we implement the generator network as a 2D U-Net in order to preserve finer details of the input image. Note that removing skip connections would give SIT an unfair advantage since SIT’s output layer has access to the original image. The bottom layer of the U-Net is reshaped into a 50-dimensional latent vector. We concatenate $\tilde{y}-y$ to the latent vector, and also concatenate it as new channels to two other feature maps in the decoder branch. All networks have four spatial resolutions, with 128 channels at the lowest resolution. In RGAE, the regressor has a simple VGG-like architecture with three down-sampling blocks. CAAE and IPGAN use this same architecture for the discriminator. In StarGAN, the discriminator and regressor share three down-sampling blocks, then split into fully connected layers of the appropriate dimension (1 output for the discriminator, m outputs for the regressor).

Batch normalization and ReLU activation is applied after all convolutional layers. The generators use max pooling and bilinear upsampling. We use He initialization [42] for convolutional layer weights. All networks are trained with Adam optimizers (moving average parameter $\beta_{1}=0.5$) for up to 10K iterations with a minibatch size of 8. The generators, discriminators and regressors are trained with a learning rate of 10^−3^, except CAAE which trains the discriminators with learning rate 10^−4^. The generator is regularized with the L1 norm of the intensity transform (difference map):

(15) $${ℒ}_{G,SIT}={ℒ}_{G}+\lambda_{\Delta x}∥\Delta x{∥}_{1},$$

where we choose $\lambda_{\Delta x}=10$.

## III. Evaluation

### A. Data

We perform image-to-image translation on research scans from the Alzheimer’s Disease Neuroimaging Initiative (ADNI) database (adni.loni.usc.edu) and clinical quality MRIs of stroke patients from the MRI-GENetics Interface Exploration (MRI-GENIE) study [39]. The longitudinal scans in ADNI enable us to assess the model’s ability to predict aging trajectories, while the stroke cohort allows us to test the model’s performance on a small dataset of lower quality scans, as well as its ability to generalize to different clinical sites.

#### 1) ADNI:

We perform image-to-image translation on longitudinal T1-weighted MRIs from ADNI conditioned on age, baseline diagnosis, and two cognitive scores: mini-mental state examination (MMSE) and clinical dementia rating (CDR). The diagnostic categories are control, mild cognitive impairment, or Alzheimer’s disease, encoded as −1, 0, and 1 respectively. MMSE ranges from 0 to 30, and lower scores indicate more severe dementia. CDR is a 5 point scale that increases with the degree of cognitive impairment. Age, MMSE and CDR are each normalized so that their respective empirical distributions in the training set have zero mean and the standard deviation of 1.

The training set consists of 3228 scans. Of these, 77 scans are from subjects with only a single timepoint scan, and the remaining 3151 are from 609 subjects with multiple timepoints (5.2 scans on average, separated by 0.79 years on average). The test set consists of 749 scans from 149 subjects with multiple timepoints (4.7 scans on average, separated by 0.81 years on average). The subjects in the training and test sets do not overlap. All model hyperparameters are tuned using only the ADNI training set.

Each scan is preprocessed with resampling to 1mm isotropic voxels, affine spatial normalization using FreeSurfer [43], and cropping to 224 × 192 slices. The 15 middle axial slices of each subject are used. During training, the images are augmented using horizontal flips, random affine transformations, and intensity rescaling.

#### 2) MRI-GENIE:

In the MRI-GENIE study, axial brain fluid-attenuated inversion recovery (FLAIR) MRIs are obtained within 48 hours of symptom onset for acute ischemic stroke. After excluding repeat scans and scans with extreme artifacts, we have 1821 subjects from 12 clinical sites each with a single FLAIR scan. 418 images acquired from the largest site are used for hyperparameter tuning (5-fold cross validation with 334 training scans and 84 validation scans). Each model is trained on 334 images from this site and tested on the 1403 scans from the 11 held out clinical sites. Age is available for all patients, and stroke severity (measured on a scale from 0–36 called NIHSS) is available for 746 patients.

Compared to ADNI, MRI-GENIE contains brain scans with much more heterogeneity and artifacts due to the acute clinical setting and various scanners used to acquire them. Many scans feature severe motion artifacts and partial volume effects, as well as large anatomical variation and/or prior disease. Examples of various artifacts are shown in Appendix Figure 7.

MRIs are preprocessed with resampling to isotropic 1mm resolution (native resolution was around 1mm × 1mm × 6mm), N4 bias field correction, ANTS registration to a FLAIR atlas [44], normalization of the white matter intensity, and cropping to 224 × 192. The 15 middle axial slices of each scan are used, and all slices from the same scan are grouped into the same validation fold. Age and stroke severity values are scaled so that their respective empirical distributions in the training data had zero mean and the standard deviation of 1. The images are also augmented using horizontal flips, random affine transformations and intensity scaling.

### B. Evaluation Metrics

We evaluate our model based on two criteria: the realism (fidelity) of the generated images, and how accurately the attributes of those images match the desired values.

#### 1) Image Fidelity Metrics:

In ADNI, the longitudinal scans in the dataset enable us to directly compare the outputs of our model to the ground truth. For every subject, we randomly select up to 5 pairs of timepoints. For each pair, we use the most central slice of the earlier scan as input to our trained model, which then predicts the central slice of the later scan. We compare the model output to the actual scan obtained at this later timepoint, using root mean square error (RMSE) and structural dissimilarity (DSSIM) [45]. RMSE is a pixel-wise metric while DSSIM compares images based on their patch statistics – the DSSIM of identical images is 0, and the DSSIM of images in which every patch is uncorrelated is 0.5. In order to better differentiate models that do not adequately change the input image, we only compute RMSE and DSSIM for pairs separated by at least one year.

In MRI-GENIE, each patient has one scan and the generated images represent counterfactuals rather than predicted (unobserved) trajectories. Since paired ground truth images are not available, we use distributional metrics to quantify the fidelity of the generated images. We compute the Fréchet Inception Distance (FID) [46] between the distribution of generated images and the distribution of real images. We also use Precision and Recall for Distributions (PRD) [47] to compute the precision (*F*_1*/*8_) and recall (*F*_8_) of our generator. A high precision indicates that most modes of the generated distribution also belong to the true distribution, whereas a high recall suggests that most modes of the true distribution belong to the generated distribution. Modes are estimated by finding clusters of images in Inception v3 embedding space.

#### 2) Attribute Matching Metrics:

Because image fidelity metrics do not distinguish between errors from target domain mismatch and those from artifacts, we also assess whether generated images match the target age. For both ADNI and MRI-GENIE datasets, we fine-tune a Inception v3 regressor (pre-trained on ImageNet classification [48]) to estimate the patient’s age from their scan. We then run this regressor on generated images and measure the similarity between the estimated age and the desired age. We report the difference between these values in years as the **AgeError**. The age error of this regressor on real test set images is −1.5 ± 5.2 years (mean and standard deviation) in ADNI, and −1.4±9.4 years in MRI-GENIE. This Inception v3 regressor is used only for evaluation, and is different in architecture from the regressor used in training of StarGAN. We deliberately use different regressors for training and evaluation, in case the generator had learned to exploit weaknesses of the particular regressor with which it was co-trained.

### C. Transform Ablations

We perform an ablation study to investigate the effect of the spatial and intensity transforms in isolation. We test the following parameterizations of the generator:

#### Base:

Our baseline is the unconstrained network based on StarGAN, which directly synthesizes an image from the generator network.

#### IT:

The generator’s output is parameterized as an intensity difference map that is added to the input image to produce an output image. As with SIT, we penalize the L1 norm of the difference map to encourage sparsity.

#### ST-Disp:

The displacement-based spatial transform constrains the generator to output deformations of the input image in terms of a displacement field *F*, similar to networks used to predict optical flow [49]. We penalize the discrete total variation norm [50] of the displacement field to encourage smoothness:

(16) $$\|F{\|}_{\text{TV}}=\frac{1}{\left|\Omega \right|}\sum_{\omega \in \Omega }\|\nabla F(\omega ){\|}_{2},$$

where $∥\nabla F(\omega ){∥}_{2}$ is approximated using finite differences. The generator loss becomes:

(17) $${ℒ}_{G,ST-Disp}={ℒ}_{G}+\lambda_{F}{∥F∥}_{TV},$$

where we choose $\lambda_{F}=1$.

#### ST-Diff:

The diffeomorphic spatial transform is constrained to output smooth deformations of the input image in terms of a stationary velocity field. The diffeomorphism enforces smoothness in the spatial transform without requiring additional regularization. It corresponds to SIT with $\lambda_{\Delta x}=\infty$.

#### SIT-Disp:

The displacement-based spatial-intensity transform outputs a displacement field and an intensity difference map. We penalize the total variation norm of the displacement field and the L1 norm of the difference map.

#### SIT-Diff (SIT):

Our method (diffeomorphic spatial-intensity transform) outputs a velocity field and an intensity difference map. Only the L1 nom of the difference map is regularized.

## IV. Experiments

For all experiments in this section, we train the models and tune hyperparameters on the training and validation sets, and report results on a separate test set that has no patient and/or site overlap with training/validation.

### A. ADNI Results

#### 1) Baseline vs. SIT Models:

For all four models, adding spatial-intensity transforms markedly improves performance on all metrics (Table I). RGAE performs poorly on trajectory matching relative to the other models, as it lacks a discriminator to keep translated images on the image manifold. Following the guidance of its own regressor prevents the model from matching the Inception v3 regressor, leading to high age error as well. SIT-RGAE’s regularized parameterization gives it a significant boost in all metrics, but without a discriminator it still fails to generate convincing outputs. StarGAN outperforms CAAE and IPGAN, as StarGAN is directly guided by a regressor to produce images matching the desired age, whereas CAAE and IPGAN rely on implicit signals from the conditional discriminator. This suggests that regressor guidance and discriminator guidance (*i.e*. adversarial training) are both beneficial for training this kind of generator.

Qualitatively, SIT-StarGAN appears to match longitudinal trajectories more closely than the unconstrained version. In Figure 2, the unconstrained generator creates an unnatural, bulging effect that erases tissue surrounding the ventricles, whereas SIT-StarGAN widens the ventricles more naturally as reflected in the ground truth. Additional qualitative results for each model and its SIT variant can be found in Appendix Figure 9.

#### 2) Transform Ablations:

Table II reports ablation study results. Both spatial transform networks attain strong performance on trajectory matching, as their parameterization prevents them from introducing spurious intensity changes, and longitudinal brain scans tend to be dominated by morphological changes. However, they perform poorly on mean age regressor error, as they cannot capture relevant intensity changes such as darkening of the gray and white matter relative to the skull. The intensity transform network and SIT-Disp models perform worse on trajectory matching than SIT-Diff, but can match age similarly well. Thus, SIT-Diff performs the best overall, even if it may be more susceptible to spurious intensity changes than spatial transforms alone. Additional qualitative results from each parameterization can be found in Appendix Figure 10.

### B. MRI-GENIE Results

#### 1) Baseline vs. SIT Models:

**Fig. 3.**
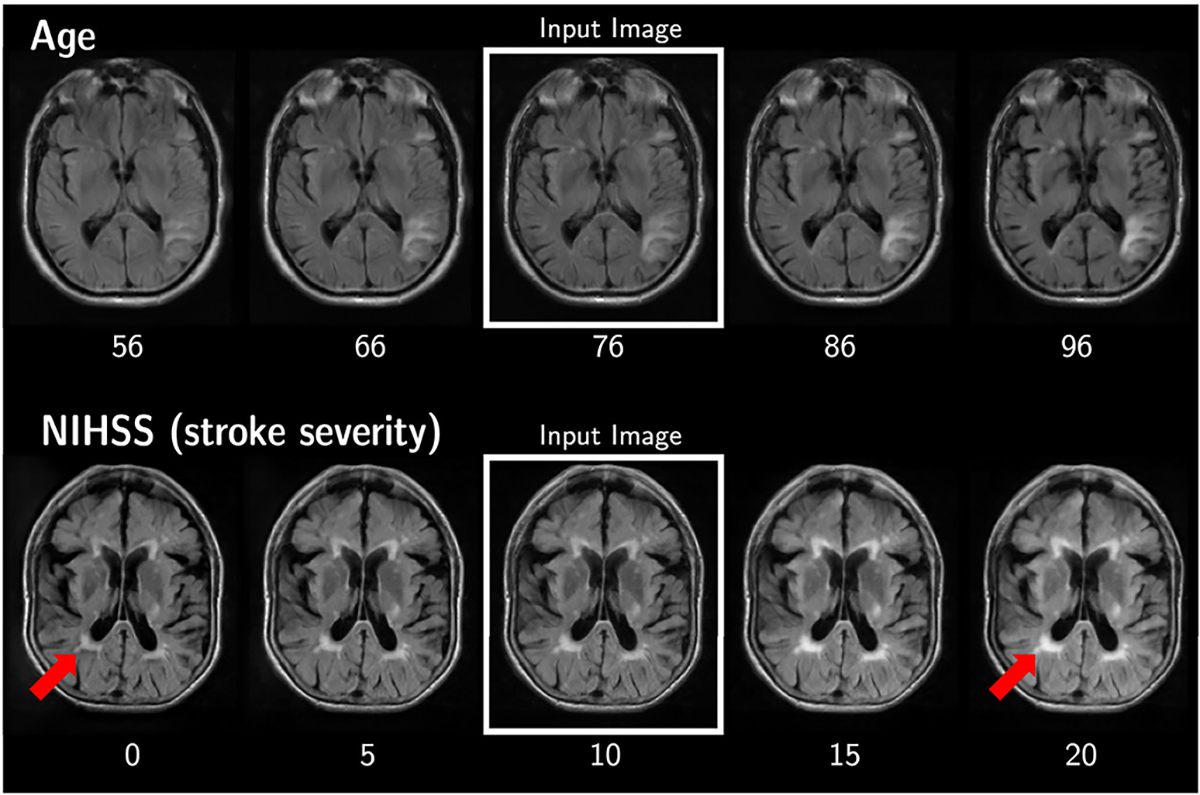
Synthetic MRIs generated by SIT-StarGAN, conditioned on two subjects’ scans in MRI-GENIE *(middle column)*. The generator transforms them into their neighboring images by conditioning on changes in age *(top row)* and stroke severity *(bottom row)*.

**Fig. 4.**
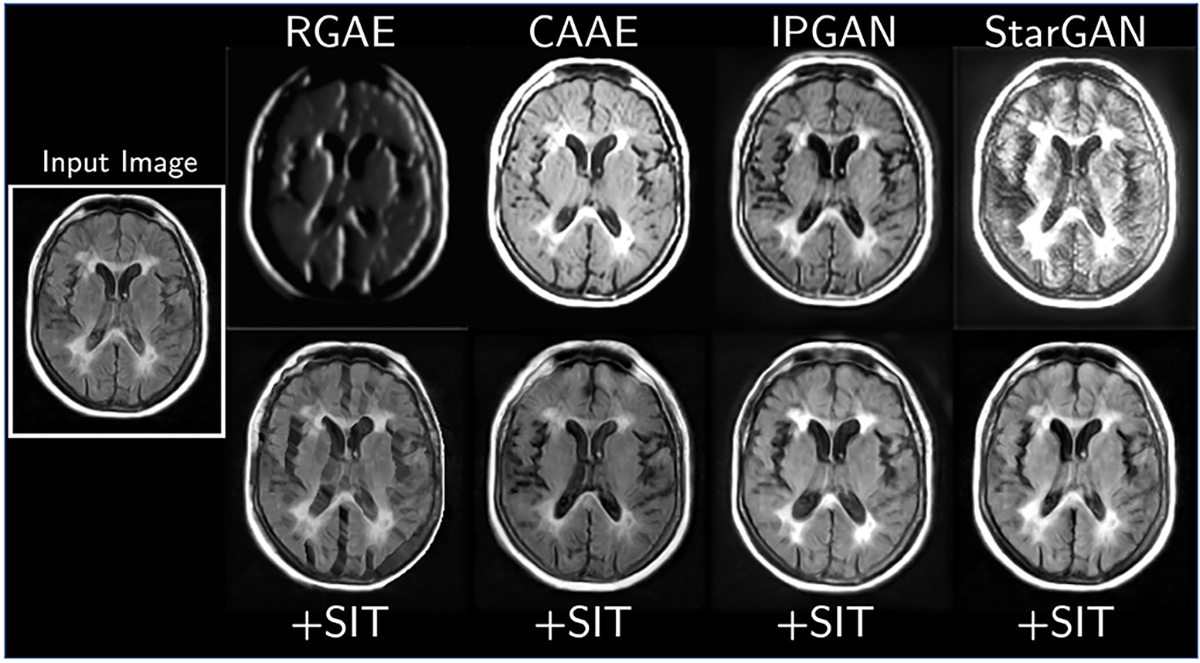
A scan from MRI-GENIE translated to a different age (originally 67 years old, translated to 82 years old) using four unconstrained models *(top row)* and their SIT variants *(bottom row)*.

The SIT variants of all four models significantly outperform their unconstrained baselines on most image fidelity and age matching metrics (Table III). Qualitatively, our SIT generators replicate known physiological patterns: that age correlates with increasing ventricular volume and widening of the sulci, and stroke severity correlates with increasing volume of white matter hyperintensities around the ventricles (Figure 3). Meanwhile, the unconstrained models introduce artifacts into translated images. RGAE performs the worst among these models, as it has not learned to create realistic images (Figure 4). CAAE preserves local image characteristics but appears unable to control the global tissue intensity. IPGAN introduces blurring and other local distortions, although the regularization inherent in the identity-preserving loss prevents it from creating strong intensity-based artifacts like the other models. StarGAN creates unnaturally bright spots throughout the tissue. SIT avoids almost all of these artifacts, and with the exception of SIT-RGAE, the models do not generate images with any prominent distortions.

#### 2) Transform Ablations:

**Fig. 5.**
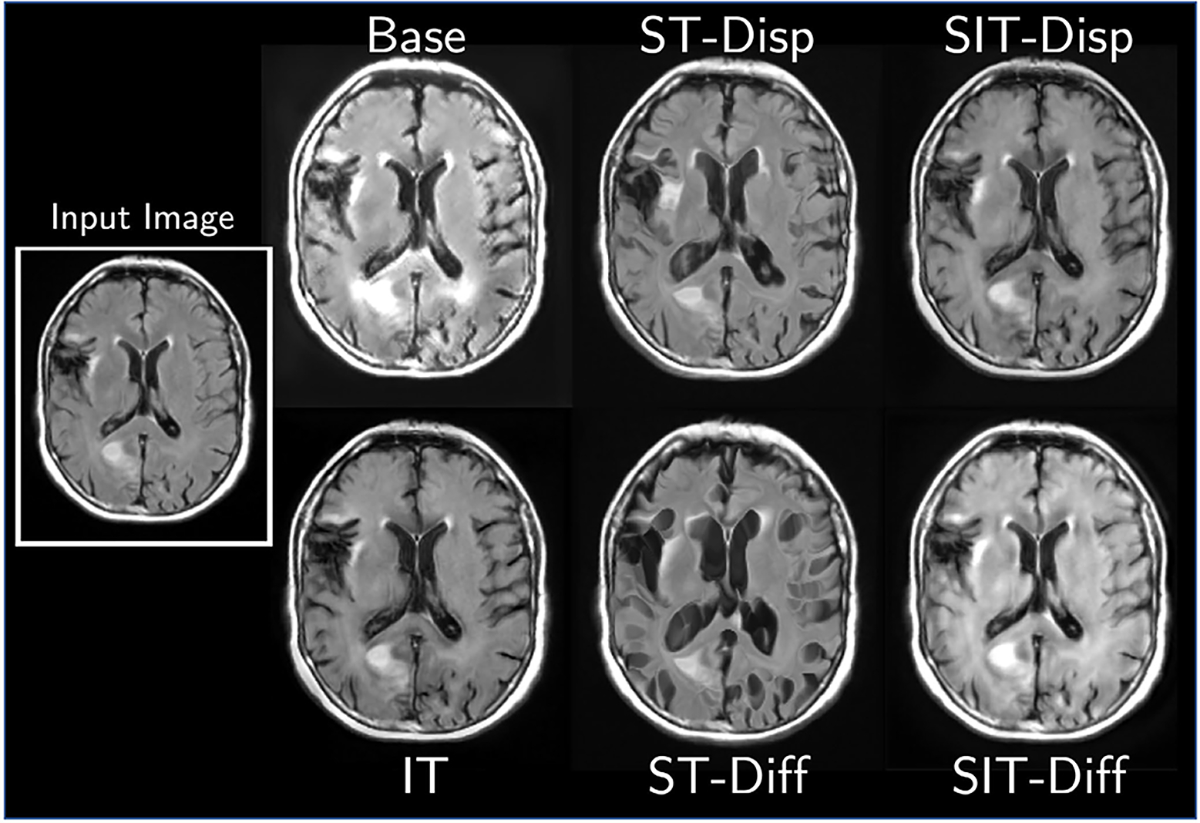
A scan from MRI-GENIE translated to a different age (originally 59 years old, translated to 84 years old) using different parameterizations of the generator in StarGAN.

Table IV reports the ablation study results. In contrast to ADNI, the spatial transform models perform poorly here, introducing unrealistic distortions to the ventricles and sulci. They are perhaps more susceptible to the high heterogeneity and diverse contrasts in MRI-GENIE, leading them to overcompensate. The intensity transform and SIT-Disp models outperform the unconstrained baseline, and are competitive with SIT-Diff, outperforming slightly on image fidelity metrics but underperforming on age error, suggesting that perhaps they are overly conservative with their transformations. In particular, good scores on FID and precision/recall can be achieved by producing images that are nearly identical to the input image without considering the target age, and we observed that these ablated models often make fewer changes to the input image than expected, resulting in the target age mismatch. In Figure 5, the baseline StarGAN produces some discontinuity artifacts around the right sulci, blurring around the upper sulci, and some artificially bright spots throughout the gray matter. There are prominent distortions in the generators parameterized by a spatial transform only, as they perhaps overcompensate for their inability to create intensity changes indicative of age differences. The intensity transform generator and the two generators parameterized with spatial-intensity transforms do not inject artifacts, although the diffeomorphic SIT is better able to simulate the growth of white matter hyperintensities near the ventricles, which is a known correlate of age in stroke patients. Thus SIT-Diff achieves the best overall results in producing realistic changes in input images.

### C. Visualizing Spatial-Intensity Transforms

**Fig. 6.**
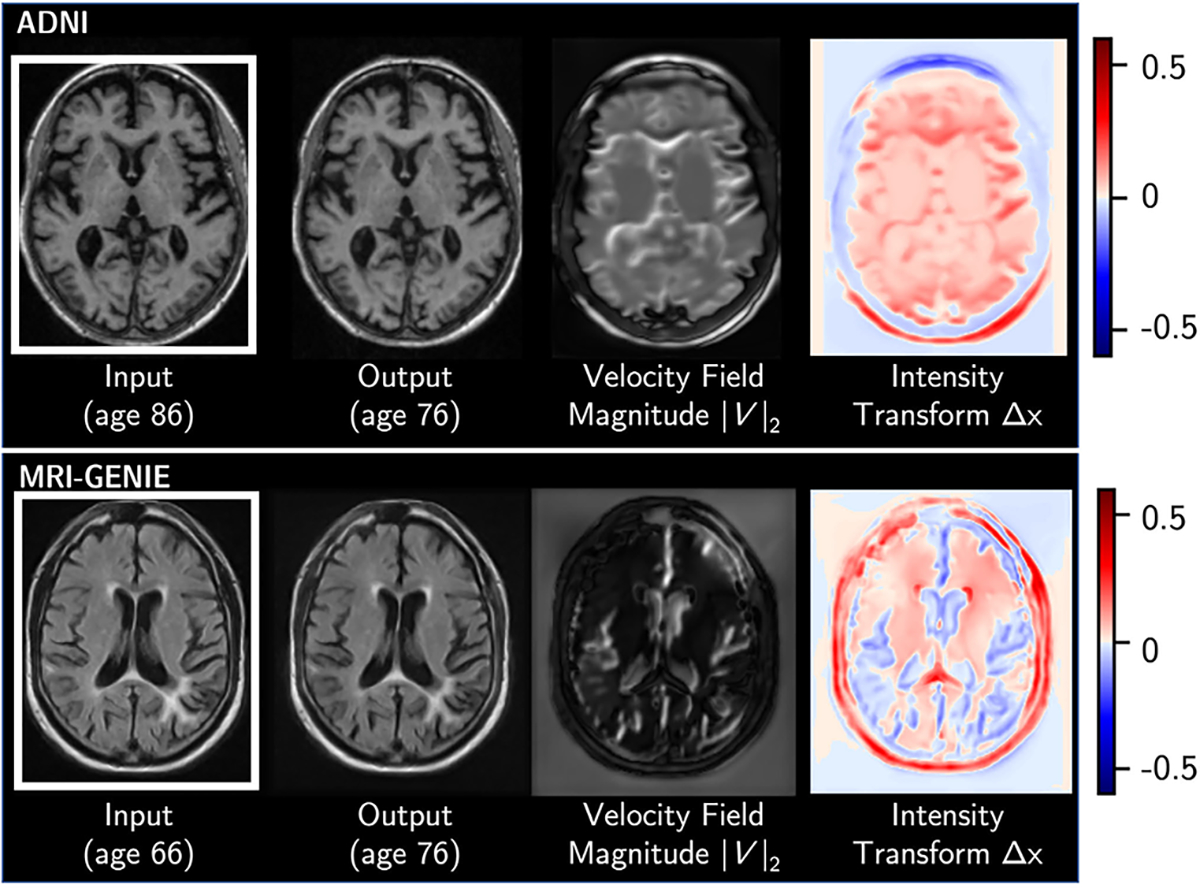
An example of the spatial and intensity transforms produced by SIT-StarGAN for an age-conditioned translation in ADNI *(top)* and MRI-GENIE *(bottom)*.

The deformation field and intensity difference map used in SIT-StarGAN are not only good priors for modeling image translation, but also provide a way to visualize distinct biological changes. In our datasets, the spatial transform highlights changes in morphology associated with age, while the intensity transform highlights changes in tissue or skull intensity (Figure 6). In the ADNI example, expansion of the ventricles and sulci with age manifests in large velocities around their borders, and the intensity difference map shows global brightening of the white matter. In MRI-GENIE, the expansion of the ventricles and sulci is also well captured by the spatial transform, while white matter hyperintensities and other tissue appearance changes are reflected in the intensity transform. These changes are fairly subtle when comparing the generated image directly to the input image, but become apparent with this visualization.

These effects, which are mixed in the representation with an unconstrained generator, can be visualized separately with SIT generators. This ability to disentangle can be valuable for finding and visualizing biomarkers or other changes that are not immediately apparent from the generated image.

## V. Discussion

### Incorporating paired training data:

In the specific application of longitudinal image prediction, it is likely that identifying true pairs during training would further improve a model’s performance over an unpaired approach. With paired training data, the translated images can be compared directly to the ground truth, providing valuable information during training. If both unpaired and paired training data exist, the model can be trained using both approaches in succession or simultaneously. We use a strictly unpaired training scheme on ADNI in order to demonstrate the generalization of our models from research scans to low-quality clinical scans without hyperparameter tuning. Moreover, most applications in medical image-to-image translation do not have access to paired data. In future work it may also be helpful to conduct a reader study with trained neuroradiologists in order to assess whether generated trajectories are sufficiently accurate and useful for clinical applications.

### Choices of network architecture:

It has been demonstrated extensively that skip connections and multi-scale structures are central features of effective image-to-image translation architectures. Indeed, we modified the architecture of the conditional adversarial autoencoder and identity-preserving GAN to include some skip connections, as the output quality degraded significantly without them. Therefore, it is fairly likely that many different backbones with these two design requirements, such as U-Net, V-Net, ResUNet [51], and Feature Pyramidal Networks [52], will all be adequate for this task, but that a simple encoder-decoder architecture may fail in the absence of more complex training schemes such as progressive growing. We conjecture that transformer-based generators, autoregressive [53] or flow-based generators [54] can be designed to produce separate intensity and spatial transforms in image-toimage tasks, but it would be challenging to adapt for denoising diffusion models [55], since it would be unclear how to model the diffusion process along spatial and intensity components simultaneously.

### Application to other organs and modalities:

Beyond brain MRIs, SIT can be used in image-to-image translation tasks involving other organs and disease processes. Indeed, the prior of a diffeomorphic transform coupled with sparse intensity changes is widely applicable to many types of phenotypic variation in CT and MRI sequences. Our framework for parameterizing generative models is not limited to the particular spatial-intensity transform we presented, and can be modified to best fit the dataset and task of interest. The diffeomorphic spatial transform can be relaxed to a displacement field if the translated image is not expected to preserve tissue topology. In addition, the regularization on the intensity transform can be modified to fit other priors. While our choice to penalize the L1 norm leads to sparse changes in intensity, some attributes may call for smooth global changes in intensity, in which case the total variation norm could be penalized. If segmentations are available, one could assign intensity priors separately to different tissues, or simply encourage intensity changes to be smooth within segmentations but not across them. Specifying intensity and spatial priors through the right parameterization of the generator can be particularly useful in image-to-image translation tasks on diseases without a standard coordinate frame such as lesions, abscesses, and aneurysms, as these types of images could be particularly challenging for generative models to train on.

## VI. Conclusion

Spatial-intensity transforms are a simple and effective technique for improving image fidelity and robustness to artifacts in generative models for medical image-to-image translation. We demonstrate SIT on two tasks and four different models. In ADNI, our SIT-based models successfully predict longitudinal T1-weighted brain MRIs from unpaired data. On a challenging dataset of clinical quality MRIs of stroke patients from multiple clinical sites, SIT outperforms unconstrained networks on image fidelity metrics without compromising their ability to match the desired attributes. The generated scans clearly capture the correlation between age and ventricle expansion, as well as between the volume of white matter hyperintensities and stroke severity. SIT networks additionally provide a disentangled view of changes in anatomical shape and tissue appearance through the velocity field and intensity difference map respectively.

SIT may be a valuable tool to visualize morphological and textural variation of organs or radiological findings conditioned on patient phenotype. By conditioning on different patient attributes such as disease status, severity, and outcome, robust image-to-image translation may help clinical researchers investigate and visualize imaging biomarkers. The development of robust conditional GANs is particularly crucial in the context of the unpredictable ways that such models can induce artifacts, as well as the need for reliable and reproducible methods in clinical research and practice.

## Figures and Tables

**Fig. 1.**
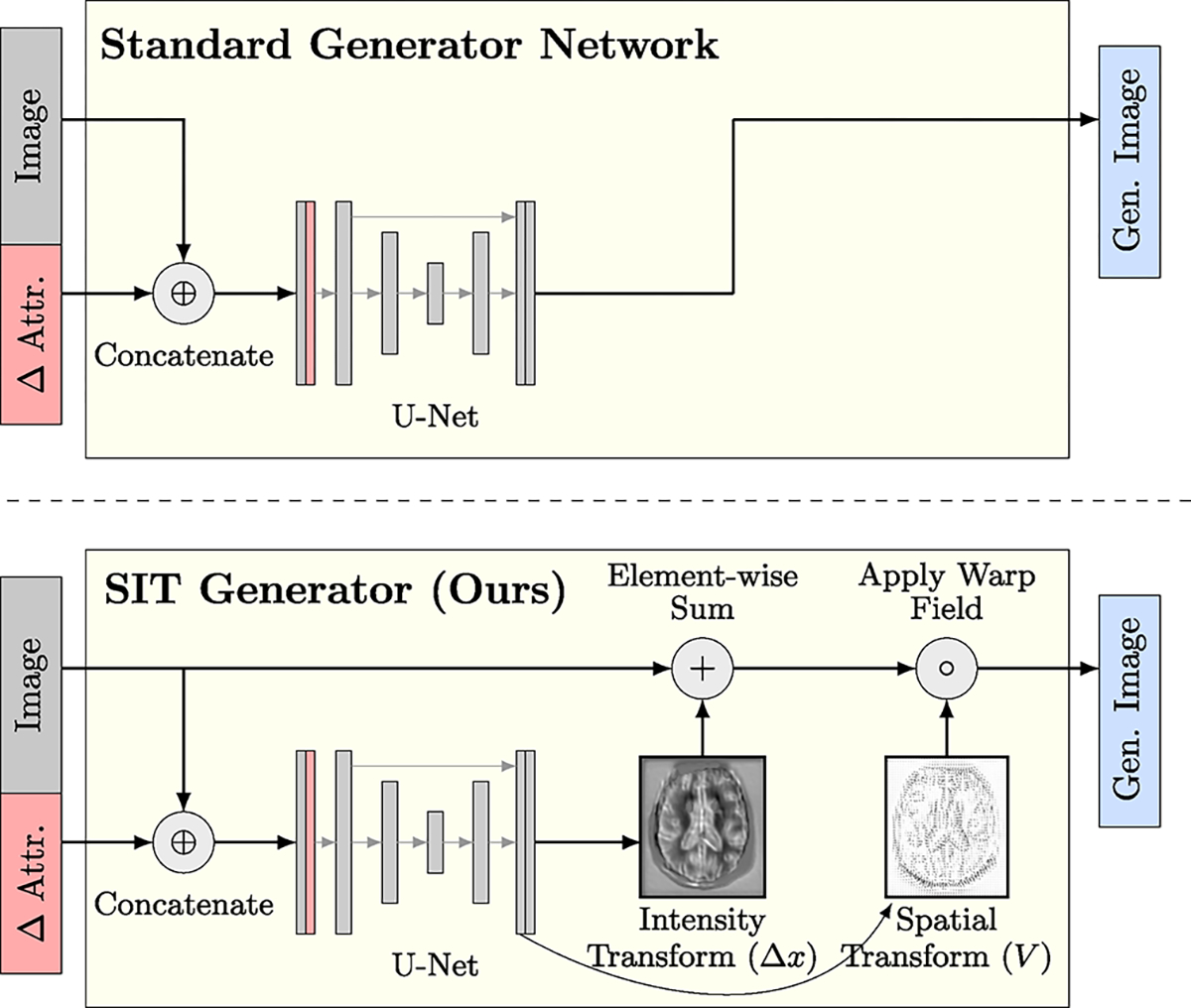
*(Top)* In most previous approaches, a generator outputs a new image directly, given an input image and desired change in attributes. *(Bottom)* Our SIT generator obtains a new image by applying an intensity difference map and smooth deformation to the input image. The intensity transform Δ*x* is regularized to be sparse and the spatial transform is a stationary velocity field *V* corresponding to a diffeomorphism, resulting in robust behavior. This figure illustrates channel-wise concatenation of the image with the target attributes, although the attributes can also be introduced in other areas of the network.

**Figure:**
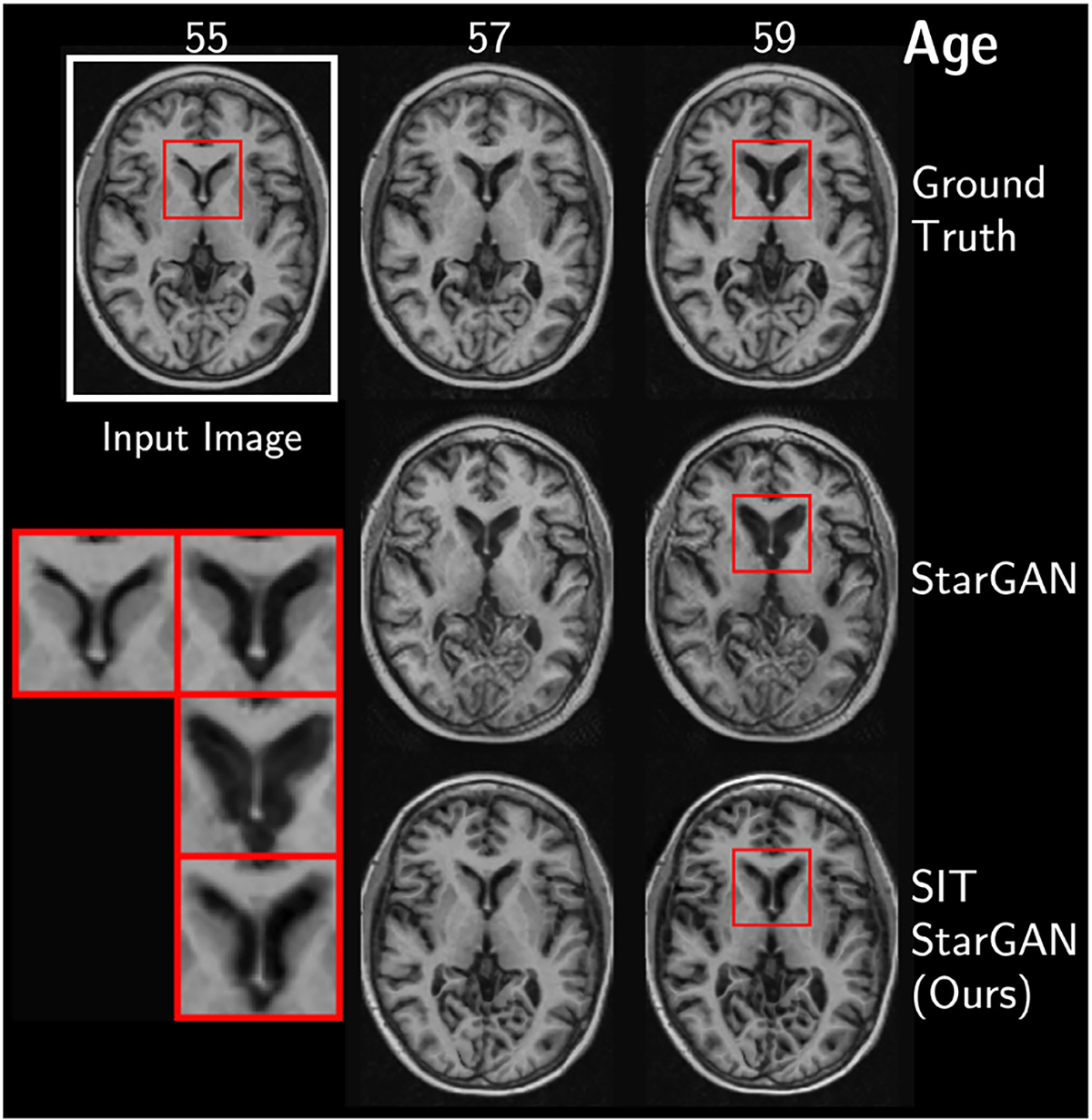
Fig. 2. *(Top Row)* True longitudinal MRIs from a subject in ADNI with mild cognitive impairment. *(Middle Row)* Predicted MRIs from an unconstrained StarGAN. *(Bottom Row)* Predicted MRIs from StarGAN with spatial-intensity transforms. *(Inset)* Adding SIT improves the prediction of ventricular growth rate.

**Fig. 7.**
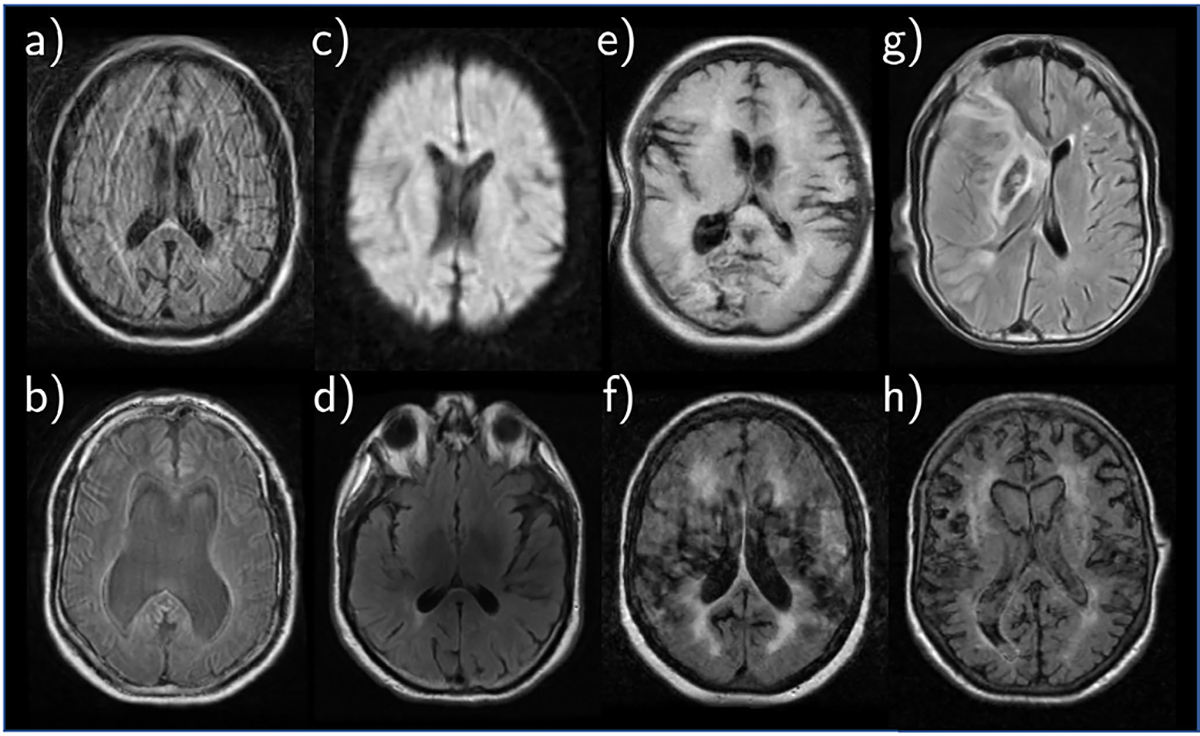
Example T2-FLAIR scans from the MRI-GENIE dataset illustrating (a) severe motion artifact, (b) partial volume effect, (c) blurriness and poor contrast, (d) bias field, (e) wraparound artifact, (f,g,h) large anatomical variation and/or prior disease.

**TABLE I:** Evaluation Metrics for Longitudinal MRI Prediction and Age Matching in ADNI Using Four Unconstrained Models and Their SIT Variants. We Report Standard Deviations Over the Test Set. We Bold the Age Error With Smallest Absolute Mean, Although One May Prefer to Tradeoff Bias for Lower Variance

Model Type	RMSE	DSSIM	Age Error
RGAE + SIT (ours)	0.26±0.02 **0.13±0.03**	0.36±0.02 **0.13±0.04**	10.26±7.44 **−2.45±6.50**
CAAE [29] + SIT (ours)	**0.14±0.02** **0.14±0.03**	0.16±0.04 **0.15±0.04**	−4.31±5.61 **0.81±7.24**
IPGAN [30] + SIT (ours)	0.13±0.02 **0.12±0.03**	0.16±0.04 **0.13±0.04**	1.90±6.12 **−1.45±5.62**
StarGAN [32] + SIT (ours)	0.16±0.03 **0.11±0.03**	0.17±0.04 **0.13±0.04**	0.82±6.54 **−0.40±6.84**

**TABLE II:** Evaluation Metrics for Longitudinal MRI Prediction in ADNI for Different Parameterizations of the Generator Applied to StarGAN

Transform	RMSE	DSSIM	Age Error

Base	0.16±0.03	0.17±0.04	0.82±6.54
IT	0.12±0.03	0.15±0.04	−0.43±7.57
ST-Disp	**0.11±0.03**	0.12±0.04	−1.92±5.59
ST-Diff	**0.11±0.03**	**0.11±0.05**	−1.80±6.06
SIT-Disp	0.15±0.03	0.18±0.04	0.68±6.33
SIT-Diff	**0.11±0.03**	0.13±0.04	**−0.40±6.84**

**TABLE III:** Evaluation Metrics for Image Fidelity and Age Matching in MRI-GENIE Using Four Unconstrained Models and Their SIT Variants. FID = Fréchet Inception Distance, P and R = precision (*F*_1/8_) and Recall (*F*_8_) as Defined in [47]. Since Distributional Metrics are Computed Once Over the Entire Test set, No Standard Deviation is Reported for Those Columns

Model Type	FID	P	R	Age Error
RGAE + SIT (ours)	54.49 **40.92**	0.00 **0.01**	**0.00** **0.00**	**4.13=1=14.75** 5.71±13.00
CAAE [29] + SIT (ours)	18.32 **4.06**	0.10 **0.80**	0.12 **0.88**	4.08±10.74 **0.83±10.66**
IPGAN [30] + SIT (ours)	11.05 **2.86**	0.14 **0.81**	0.26 **0.95**	4.06±9.96 **2.70±9.50**
StarGAN [32] + SIT (ours)	22.77 **2.07**	0.09 **0.90**	0.30 **0.97**	3.10±13.21 **1.97±12.28**

**TABLE IV:** Image Fidelity and Age Matching Metrics for in MRI-GENIE for Different Parameterizations of the Generator Applied to StarGAN

Transform	FID	P	R	Age Error

Base	22.77	0.09	0.30	3.10±13.21
IT	**0.85**	**0.98**	**0.98**	2.67±11.71
ST-Disp	2.61	0.82	0.97	6.18±11.94
ST-Diff	5.50	0.53	0.92	3.02±10.40
SIT-Disp	1.14	**0.98**	0.95	2.44±11.71
SIT-Diff	2.07	0.90	0.97	**1.97±12.28**

**Fig. 8.**
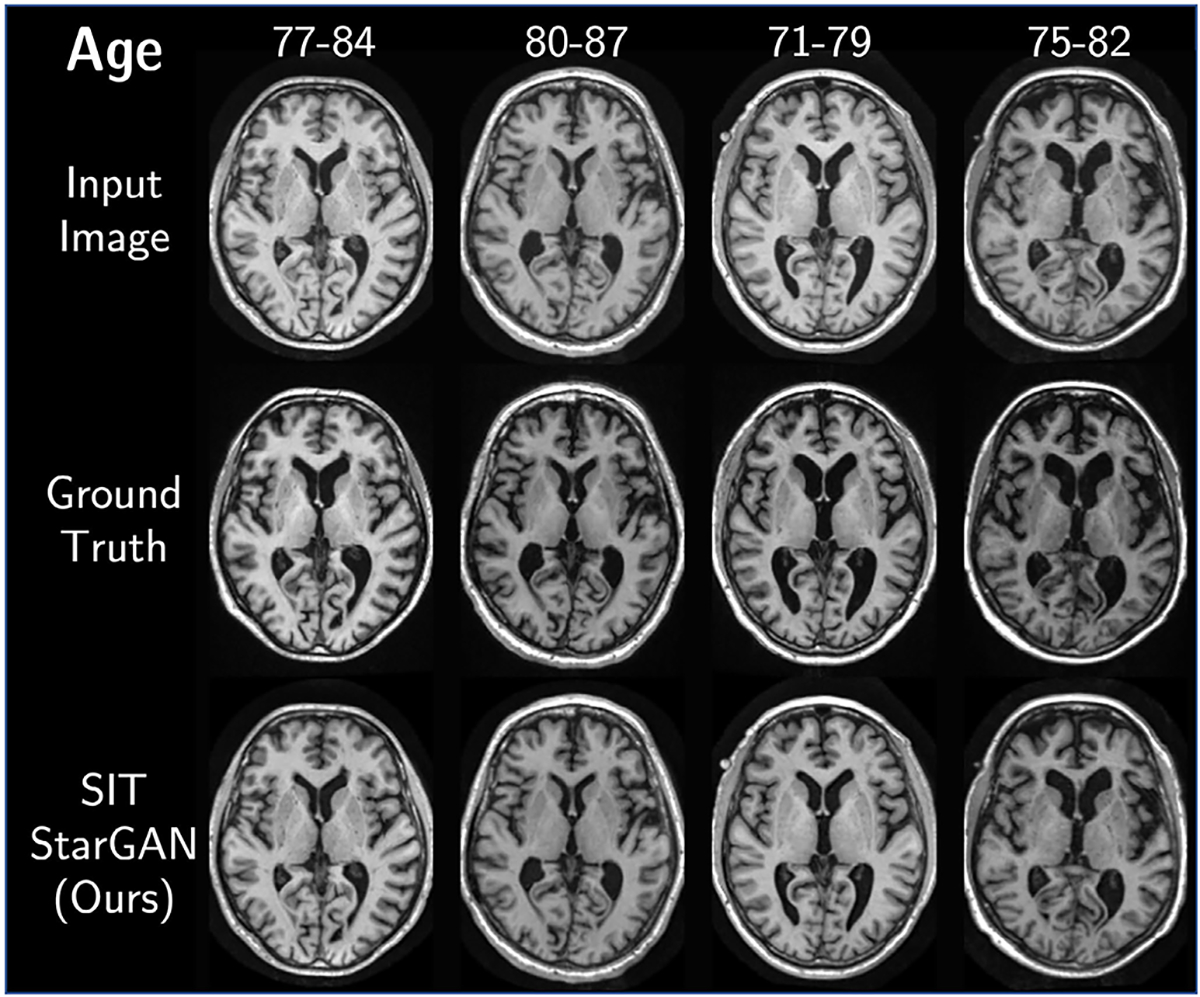
Failure cases of SIT-StarGAN on ADNI for patients with mild cognitive impairment or Alzheimer’s disease. Each column shows a different subject: earlier timepoint *(top)*, ground truth for later timepoint *(middle)*, and prediction of later timepoint *(bottom)*.

**Fig. 9.**
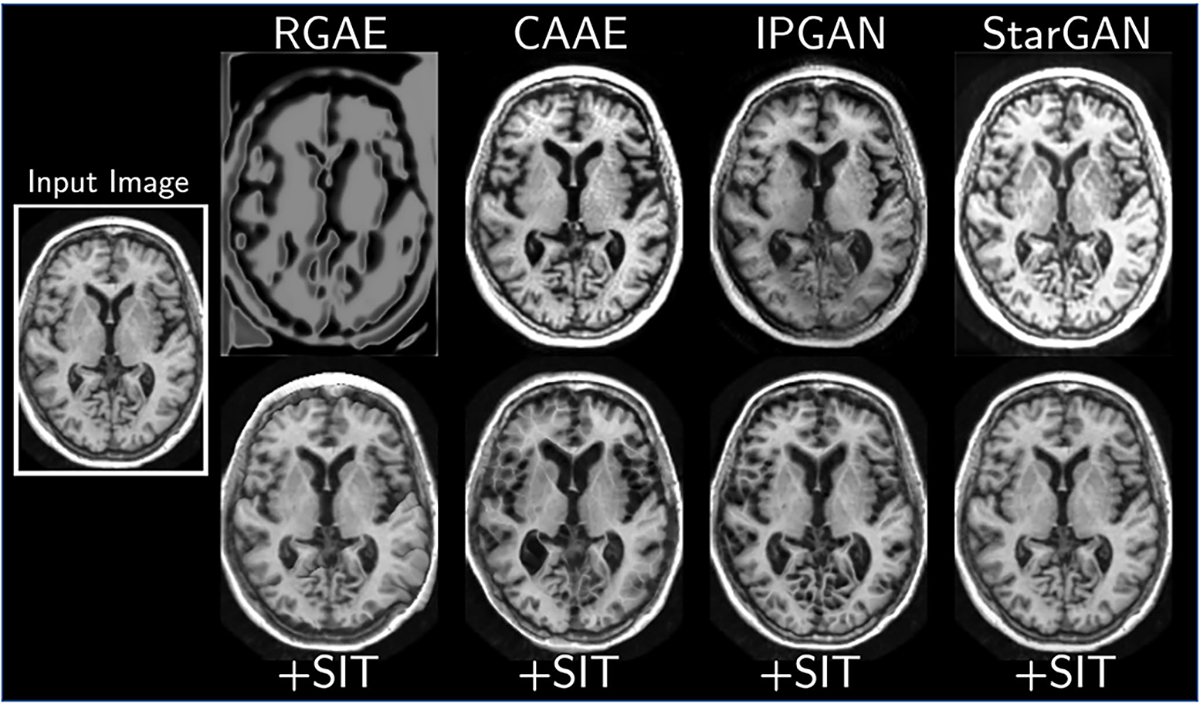
A scan from ADNI translated to a different age (originally 57 years old, translated to 72 years old) using various models and their SIT-based versions.

**Fig. 10.**
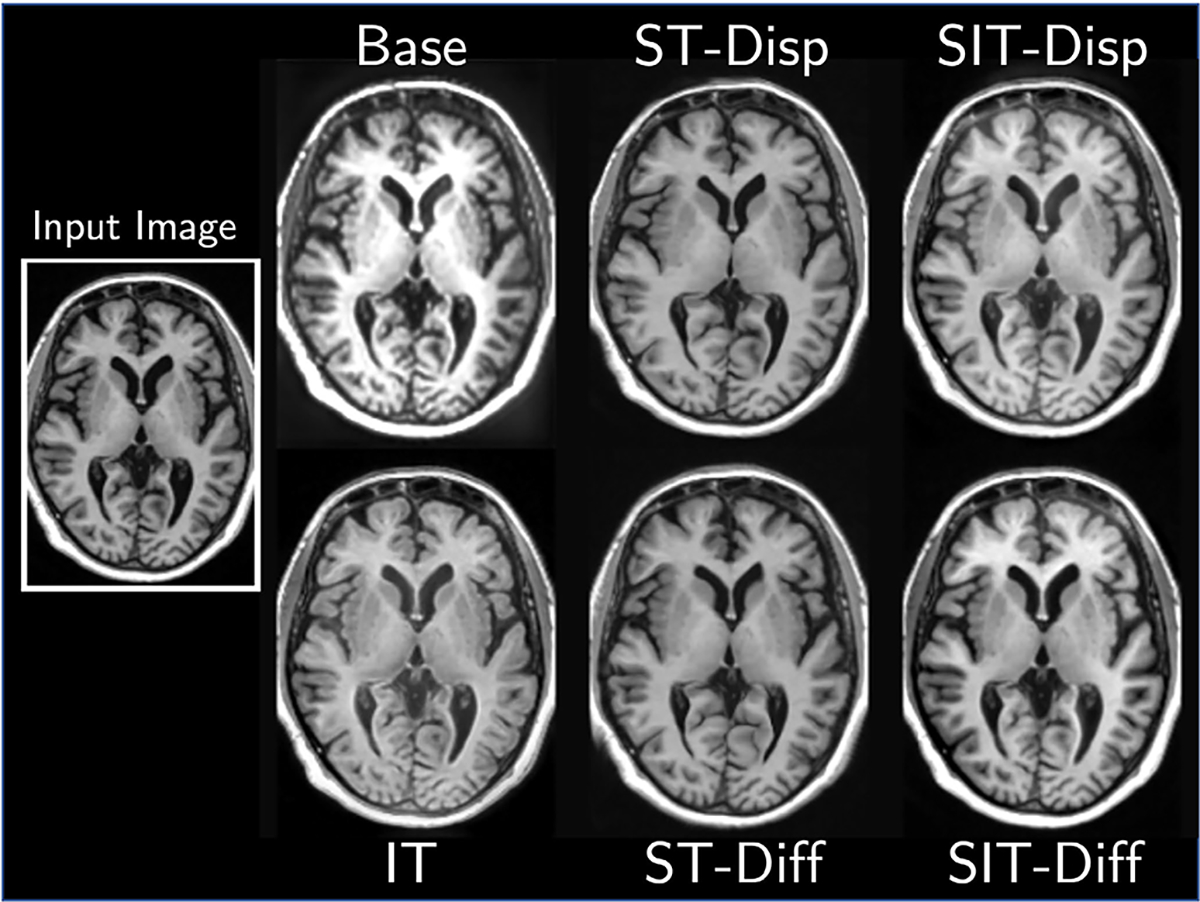
A scan from ADNI translated to a different age (originally 79 years old, translated to 64 years old) using different parameterizations of the generator in StarGAN.

## Footnotes

1 Our implementation differs in several ways. We predict intensity differences and introduce sparsity regularization, to encourage morphological changes to be captured by spatial transforms. We use a diffeomorphic spatial transform, which is more expressive than an affine or B-spline transform. Finally we share parameters between the networks predicting spatial and intensity transforms, which improves efficiency. These design choices are more suitable for our task and for generating high-fidelity images.

2 Categorical atributes can be readily included by adding a classifier to the network.

3 The Wasserstein loss improves the stability of training when compared to the original adversarial loss, as it provides a valid gradient even when the supports of the generated and real distributions do not overlap. The gradient penalty term encourages the discriminator to have gradients with norm 1 in order to make it a 1-Lipschitz function.

## Appendix

### A. Artifacts in MRI-GENIE

Since the ADNI dataset consists of high quality research scans, it contains few artifacts. In contrast, the MRI-GENIE dataset, obtained during acute care across many clinical sites, is highly heterogeneous. The T2 FLAIR scans feature severe motion artifacts, partial volume effects, blurriness, graininess, stripes, bias fields that remain after N4 bias field correction, wraparound artifacts, large anatomical variation and prior disease. The extreme variation in contrast and image appearance makes learning image-to-image translation from unpaired scans more challenging.

### B. Failure Cases

In most cases, SIT’s failure modes occur when it is overly conservative in changes to the input image. This is most easily observed in ADNI trajectories featuring rapid neurodegeneration (Figure 8). We believe this could be mitigated to some extent by conditioning the generator directly on neurodegenerative indicators (baseline diagnosis and cognitive scores) rather than differences in these attributes, although we decided not to adjust our treatment of these variables in order to make our approach more consistent and easier to grasp.

**TABLE V:** Line Search of Regularization Weight on SIT-StarGAN on ADNI. ∞ Corresponds to the ST-Diff Ablation Model

λ_Δ*x*_	RMSE	DSSIM	Age Error

**0.01**	**0.12±0.03**	0.12±0.05	**0.36±6.86**
**0.1**	**0.12±0.03**	0.12±0.04	−0.95±7.06
**1**	**0.13±0.03**	0.13±0.05	−0.75±7.23
**10**	**0.11±0.03**	0.13±0.04	−0.40±6.84
**100**	**0.11±0.03**	**0.11±0.04**	−0.69±6.88
**∞**	**0.11±0.03**	**0.11±0.05**	−1.80±6.06

SIT is still highly susceptible to artifacts on RGAE. Although we do not observe obvious artifacts in outputs of SIT-CAAE and SIT-IPGAN, their inferior image fidelity metrics relative to SIT-StarGAN suggest that combining adversarial training with regressor guidance is valuable for building a robust image-to-image translation model. Still, we do not claim that StarGAN is a fully optimized architecture for this task, and future work could explore incorporating more recent developments in the generative modeling literature, which are orthogonal to SIT.

### C. Effect of Regularizer Weight on SIT

We conduct a line search of the regularizer weight determining the sparseness penalty for the intensity transform in SIT-StarGAN. We sweep $\lambda_{\Delta x}$ from 0.01 to 100. We find that the model yields reasonably strong performance at a range of values (Tables V and VI). We select $\lambda_{\Delta x}=10$ based on its good age matching scores on both datasets.

### D. Additional Qualitative Results

See Figure 10.

**TABLE VI:** Line Search of Regularization Weight on Sit-Stargan on Mri-Genie. ∞ Corresponds to the ST-Diff Ablation Model

λ_Δ*x*_	FID	P	R	Age Error

**0.01**	**0.84**	**0.97**	**0.98**	4.15±12.05
**0.1**	1.58	**0.97**	0.96	2.22±11.99
**1**	1.41	0.94	0.97	3.17±12.42
**10**	2.07	0.90	0.97	**1.97±12.28**
**100**	2.47	0.78	**0.97**	4.23±11.69
**∞**	5.50	0.53	0.92	3.02±10.40
